# Approaches for Numerical Modeling and Simulation of the Filling Phase in Injection Molding: A Review

**DOI:** 10.3390/polym15214220

**Published:** 2023-10-25

**Authors:** Markus Baum, Denis Anders, Tamara Reinicke

**Affiliations:** 1 Group for Computational Mechanics and Fluid Dynamics, Cologne University of Applied Sciences (TH Köln), Steinmüllerallee 1, 51643 Gummersbach, Germany; 2 Chair of Product Development, University of Siegen, Paul-Bonatz-Str. 9-11, 57068 Siegen, Germany

**Keywords:** injection molding simulation, filling simulation, computational fluid dynamics, filling phase, 1D-models, 2D-models, 2.5D-models, 3D-models, rheological models

## Abstract

Injection molding is a multiphase process that requires accurate simulation of the filling phase. This is a key element in predicting the complete injection molding cycle. The filling phase presents a complex set of challenges, including migrating melt fronts, multi-phase flow, non-Newtonian fluid dynamics, and intertwined heat transfer. Evolving from 1D to 2D, 2.5D, and 3D techniques, filling simulation research has adapted to capture the intricacies of injection-molded parts. However, the need for accuracy in the characterization of the rheological properties of polymers during filling is still of paramount importance. In order to systematically categorize the numerical methods used to simulate the filling phase of injection molding, this review paper provides a comprehensive summary. Particular emphasis is given to the complex interaction of multiple geometric parameters that significantly influence the dynamic evolution of the filling process. In addition, a spectrum of rheological models is thoroughly and exhaustively explored in the manuscript. These models serve as basic mathematical constructs to help describe the complex viscous behavior of polymers during the filling phase. These models cover a spectrum of complexity and include widely recognized formulations such as the Power-Law, second-order, Herschel–Bulkley, Carreau, Bird–Carreau, and Cross models. The paper presents their implementation to include the temperature-dependent influence on viscosity. In this context, the extensions of these models are explained in detail. These extensions are designed to take into account the dynamic viscosity changes caused by the different thermal conditions during the filling process. An important contribution of this study is the systematic classification of these models. This categorization encompasses both academic research and practical integration into commercial software frameworks. In addition to the theoretical importance of these models, their practical value in overcoming challenges in the field of injection molding is emphasized. By systematically outlining these models within a structured framework, this classification promotes a comprehensive understanding of their intrinsic characteristics and relevance in different scenarios.

## 1. Introduction

The filling phase is the first step in the injection molding process and is crucial for simulating the entire injection molding cycle since all subsequent cycle steps depend on the conditions from the filling phase. Generally, the filling phase is a complex three-dimensional transient problem characterized by a moving melt front, multiphase, non-Newtonian fluid flow, and coupled heat transfer problems. The results of the filling phase are used to simulate the subsequent phases (e.g., cooling and packing). They also influence the mesh model used in the subsequent simulations. Therefore, developing the process simulation highly depends on the development of the filling simulation model.

Research in the field of injection molding filling simulation has been ongoing on for several decades. The first studies date back to the 1950s. Over the years, several milestones have been reached in the field. The focus has shifted from 1D models to 2D, 2.5D, and 3D models. Originally, 1D models were used to simulate simple flow patterns such as tube, disc, and strip flow [1]. However, due to the complexity of injection-molded parts and their non-Newtonian and non-isothermal rheological behavior, 1D models cannot accurately represent the actual filling process [2]. Therefore, for the simulation of the filling phase of injection molding, 2D, 2.5D, and 3D models are considered more appropriate.

In addition to selecting the spatial resolution of the model, the accurate description of the rheological behavior of polymers during the filling phase is crucial. There are several mathematical approaches to the modeling of viscosity behavior. Common viscosity models include the Power-Law, second-order, Herschel–Bulkley, Bingham, Carreau, Bird–Carreau, and Cross models, each extended by the temperature shift factor. These models help to simulate the filling process more accurately and provide a detailed characterization of the viscosity.

Many previous publications have dealt with only partial aspects of numerical approaches, neglecting a comprehensive overall view. These publications are often limited to the description of individual numerical approaches. As a result, similar model approaches are often classified into different spatial discretizations with insufficient attention to the spatial dimension and without taking the rheology into account. However, when examined in more detail, spatial discretization becomes important. For example, the concepts of midplane, surface, and solid models are highlighted by Cardozo in [2]. But the focus on numerical implementation and integration of spatial resolution is missing. Some spatial classification is provided in [3]. However, this publication has shortcomings in the description of rheology and the classification of the 2D approach. Other works, such as [1,4,5,6], also do not sufficiently address the clear distinction between the different approaches. Commercial software and/or ongoing research activity may remain unclassified. In this context, a clear classification of the different modeling approaches is crucial in order to highlight the risks and limitations of the numerical approximation. This study is characterized by an accurate categorization of the different model approaches, which allows comparability. Furthermore, common mathematical principles of established rheological injection molding simulation models are included in the analysis to enhance research comparability. The main objective of this paper is providing an overview of the different numerical approaches and their classification. The focus is on summarising the different rheological models for the filling phase and not only on the spatial resolution of the different modeling approaches. Special attention will be given to the differentiation of these approaches both in the research field and in the commercial field.

The structure of this paper is as follows: In Section 2, a detailed overview and a clear distinction of the different numerical and rheological models will be presented. This is followed in Section 3 by a historical classification of these models, both in the research field and in the commercial field. A summary of the relevant literature sources is given, as is a classification of the models. This chapter also discusses the special characteristics of some software approaches the commercial field.

## 2. Modeling of the Filling Phase

The filling phase is a critical process step in injection molding, where the molten polymer flows into the mold cavity to form the final product geometry after it cools down. Various modeling approaches have been developed to describe this complex process, including accurate predictions of the polymer melt behavior. Different approaches of modeling the filling phase in injection molding simulations are discussed in this chapter. These approaches include 1-dimensional, 2-dimensional, 2.5-dimensional as well as 3-dimensional models. Characteristic differences between these approaches will be made to ensure a precise distinction between the models. In addition, various rheological models that are relevant for the filling phase will be explained, along with their mathematical formulations. By describing these modeling approaches, valuable insight is provided to assist in selecting the most appropriate modeling approach to be used for specific molding scenarios.

### 2.1. 1-Dimensional Model

The 1-dimensional (1D) models have proven to be efficient and fast in predicting pressure and temperature distributions in injection molding processes. However, their utility is limited to simple and regular geometries such as strips, discs, fans, and tubes [3]. However, the great majority of injection-molded parts have complex 3-dimensional (3D) shapes that require a detailed consideration of the non-Newtonian and non-isothermal behavior of polymer melts. Consequently, 1D models cannot provide an accurate representation of the filling process for most mold cavities. Nonetheless, these models are useful for predicting the behavior of runner systems within the mold. While 1D models offer speed and efficiency, they are limited in their ability to provide a comprehensive understanding of injection molding processes for most molded parts [1,3].

**Figure 1:**
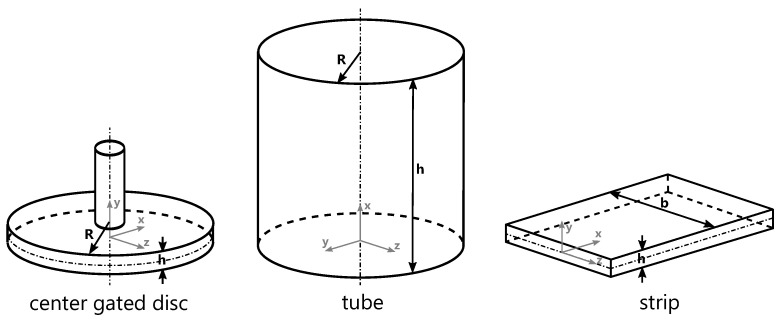
Schematic description of the 1D models: center gated disc, tube and strip.

The exploration of unidirectional models has an extensive scientific legacy tracing back to the seminal works of Kamal and Kenig [7,8], Wu et al. [9], Nunn and Fenner [10], Stevenson [11], and William and Lord [12,13]. Similar investigations of thin, rectangular cavities were conducted by Harry and Parrott [14], and Thienel and Menges [15]. These models are characterized by their 1D nature and can be described using different mathematical approximations. These mathematical approximations can be categorized into the geometric shapes of a center gated disc, tube, and strip, as shown in Figure 1.

In order to describe the mathematical approximation of the geometric shape of the center gated disc, it should be noted that in the current notation, *x* and *y* represent the streamwise and transverse coordinates, respectively, while the pressure gradient is denoted by −∂p/∂x=Λ. The variables used in these equations are as follows: *R* denotes the radial coordinate, *h* the thickness of the part, η the viscosity, θ the sector angle and *u* the velocity in flow direction. The mathematical approximation of the volume flow $\dot{V}$ with the above assumptions can be expressed in terms of

(1) $$\dot{V}=\int_{A}^{}u\left(\theta ,y\right)\;dA=\int_{0}^{2\pi }\int_{-h/2}^{h/2}u\left(\theta ,y\right)\;Rd\theta \;dy$$

In the context of the consideration of a small channel characterized by the essential aspect ratio R≫h, subject to the constraint $u\left(\theta ,y\right)\overset{!}{=}u\left(y\right)$ due to the radial symmetry and assuming no-slip at the channel walls, the volume flow in Equation (1) can be reformulated by means of partial integration as follows:

(2) $$\dot{V}=2\pi R\int_{-h/2}^{h/2}u\left(y\right)\;dy=2\pi R\int_{-h/2}^{h/2}\dot{\gamma }y\;dy$$

The shear rate $\dot{\gamma }$ for laminar plane Poiseuille flow is defined by the following equation:

(3) $$\dot{\gamma }=-\frac{du}{dy}=\frac{\Lambda y}{\eta }$$

By considering the shear rate in Equation (3) and substituting the fluidity *S* according to Equation (6), the formulation of Equation (4) is obtained:

(4) $$\dot{V}=2\pi R\Lambda \int_{-h/2}^{h/2}\frac{y^{2}}{\eta }\;dy=2\pi R\Lambda S$$

Consequently, the pressure gradient Λ and fluidity equations can be formulated as follows:

(5) $$\Lambda =\frac{\dot{V}}{2\pi RS}$$

(6) $$S=\int_{-h/2}^{h/2}\frac{y^{2}}{\eta }\;dy$$

In order to describe the mathematical approximation of the tube, it should be noted that in the current notation, *x* and *r* represent the streamwise and transverse coordinates, respectively, while the pressure gradient is denoted by −∂p/∂x=Λ. The variables used in these equations are as follows: *R* denotes the radius of the tube, η the viscosity, and *u* the velocity in flow direction. The mathematical approximation of the volume flow $\dot{V}$ with the above assumptions can be expressed in terms of

(7) $$\dot{V}=\int_{A}^{}u\;dA=2\pi \int_{0}^{R}u\left(r\right)r\;dr$$

According to the Hagen–Poiseuille law for laminar pipe flow, the shear rate $\dot{\gamma }$ is given by

(8) $$\dot{\gamma }=-\frac{du}{dr}=\frac{\Lambda r}{2\eta }$$

Taking into account the boundary condition that there is no-slip on the walls, the volume flow equation can be derived by partial integration from Equation (7), introducing the shear rate and substituting the fluidity *S* according to Equation (11):

(9) $$\dot{V}=\pi \int_{0}^{R}\dot{\gamma }r^{2}\;dr=\frac{\pi \Lambda }{2}\int_{0}^{R}\frac{r^{3}}{\eta }\;dr=\frac{\pi \Lambda S}{2}$$

Consequently, the pressure gradient Λ and fluidity equations can be formulated as follows:

(10) $$\Lambda =\frac{2\dot{V}}{\pi S}$$

(11) $$S=\int_{0}^{R}\frac{r^{3}}{\eta }\;dr$$

In order to describe the mathematical approximation of flow through a thin strip, it should be noted that in the current notation, *x* and *y* represent the streamwise and transverse coordinates, respectively, while the pressure gradient is denoted by −∂p/∂x=Λ. The variables used in these equations are as follows: *h* denotes the thickness of the part, η the viscosity, *b* the width of the part and *u* the velocity in flow direction. The mathematical approximation of the volume flow $\dot{V}$ with the above assumptions can be expressed in terms of

(12) $$\dot{V}=\int_{A}^{}u\left(y,z\right)\;dA=\int_{-b/2}^{b/2}\int_{-h/2}^{h/2}u\left(y,z\right)\;dy\;dz$$

In the context of the consideration of a small rectangular channel characterized by the essential aspect ratio b≫h, subject to the constraint $u\left(y,z\right)\overset{!}{=}u\left(y\right)$ and assuming no-slip at the channel walls, the solution of Equation (12) can be formulated as follows:

(13) $$\dot{V}=b\int_{-h/2}^{h/2}u\left(y\right)\;dy=b\int_{-h/2}^{h/2}\dot{\gamma }y\;dy$$

The shear rate $\dot{\gamma }$ for laminar plane Poiseuille flow is defined by the following equation:

(14) $$\dot{\gamma }=-\frac{du}{dy}=\frac{\Lambda y}{\eta }$$

By considering the shear rate and substituting the fluidity *S*, the formulation of Equation (15) is obtained:

(15) $$\dot{V}=b\Lambda \int_{-h/2}^{h/2}\frac{y^{2}}{\eta }\;dy=b\Lambda S$$

Consequently, the pressure gradient Λ and fluidity equations can be formulated as follows:

(16) $$\Lambda =\frac{\dot{V}}{bS}$$

(17) $$S=\int_{-h/2}^{h/2}\frac{y^{2}}{\eta }\;dy$$

A brief summary of the governing relationships for 1D volume flows with different geometry types (Figure 1) is given in Table 1.

### 2.2. 2-Dimensional Model

**Figure 2:**
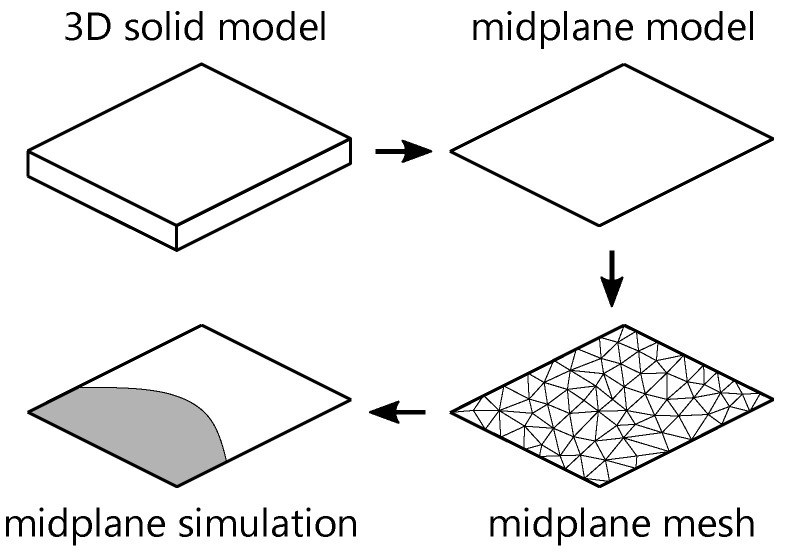
Schematic process of a midplane simulation.

In a variety of applications, injection-molded parts have complex shapes and thin-walled structures. Consequently, researchers have carried out theoretical studies, focusing on filling thin cavities. These studies used the Hele–Shaw approximation and were carried out by Richardson [16], White [17], Kamal et al. [18,19], Broyer and Tadmor [20,21,22,23] as well as Hieber and Shen [24,25,26]. Within the framework of these models, the flow is assumed to be quasi-stationary and the inertial terms can be neglected due to the low Reynolds numbers associated with the flow of molten polymers [2]. The filling of a mold cavity is represented as a 2-dimensional (2D) flow problem in which the velocity in the midplane of the cavity is related to the pressure gradient by a quantity called fluidity *S* [1]. The fluidity represents the effects of changes in the temperature and the rheology of the gap. In order to represent the 3D geometry of the part, an arbitrary planar midplane with a specific thickness is used. This model is called a midplane model and is often considered as a 3D model in an industrial context. In academic studies, the model is usually referred to as 2.5-dimensional (2.5D) [2,5,27]. In terms of the filling process and mesh representation, midplane models can be considered as 2D models, as shown in Figure 2 [28]. Figure 2 shows a schematic procedure for midplane-based simulations, starting from the 3D solid model, through the generation of the midplane mesh, to the simulation result.

**Figure 3:**
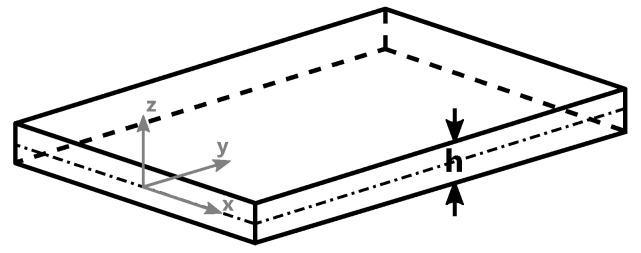
Schematic description of a Hele–Shaw configuration.

In these 2D models, the Hele–Shaw approach is used for the analysis of the non-isothermal flow of an inelastic generalized Newtonian fluid within a thin cavity of arbitrary planar shape. It is assumed that the gap thickness *h* in the *z*-direction is much smaller than the other dimensions and that there is symmetry along the midplane, as shown in Figure 3 [2,29,30].

According to Chiang and Hieber [24,31,32], the exploitation of the Hele–Shaw approximation provides simplified equations for the conservation of mass and momentum:

(18) $$\frac{\partial }{\partial x}\left(\int_{0}^{h/2}\rho u\;dz\right)+\frac{\partial }{\partial y}\left(\int_{0}^{h/2}\rho v\;dz\right)=\int_{0}^{h/2}\frac{d\rho }{dt}dz$$

(19) $$\frac{\partial p}{\partial x}=\frac{\partial }{\partial z}\left(\eta \;\frac{\partial u}{\partial z}\right)$$

(20) $$\frac{\partial p}{\partial y}=\frac{\partial }{\partial z}\left(\eta \;\frac{\partial v}{\partial z}\right)$$

(21) $$\frac{\partial p}{\partial z}=0$$

The aforementioned equations utilize h/2 to denote the half gap thickness in the *z*-direction perpendicular to the midplane, and *x* and *y* to represent the respective flow directions with their corresponding velocity components, *u* and *v*. The parameter ρ(T) represents the temperature-dependent fluid density, *t* denotes time, *p* denotes pressure, and $\eta (T,p,\dot{\gamma })$ represents fluid viscosity. The following equation describes the shear rate $\dot{\gamma }$, neglecting the *z*-direction since the Hele–Shaw formulation does not account for flow in the direction of the mold thickness [4,31].

(22) $$\dot{\gamma }=\sqrt{{\left(\frac{\partial u}{\partial z}\right)}^{2}+{\left(\frac{\partial v}{\partial z}\right)}^{2}}$$

The following boundary conditions apply to the velocity components *u* and *v* at the interface between the mold and the melt:

(23) u=v=0atz=±h/2

Taking into account the symmetry of the midplane, the following equation is derived [31,33]:

(24) $$\frac{\partial u}{\partial z}=\frac{\partial v}{\partial z}=\frac{\partial T}{\partial z}=0\;\;\mathrm{at}\;z=0$$

Since the pressure is independent of the *z*-direction, the following mathematical formulations for determining the velocity components can be obtained through partial integration of the momentum equation and taking into account the no-slip conditions [2,3,34].

(25) $$u=-\frac{\partial p}{\partial x}\int_{z}^{h/2}\frac{\tilde{z}}{\eta }\;d\tilde{z}$$

(26) $$v=-\frac{\partial p}{\partial y}\int_{z}^{h/2}\frac{\tilde{z}}{\eta }\;d\tilde{z}$$

Regarding the average velocities $\bar{u}$ and $\bar{v}$, the following mathematical description applies [34]:

(27) $$\bar{u}=-\frac{\partial p}{\partial x}\frac{S}{h/2}=\frac{1}{h/2}\int_{0}^{h/2}u\;dz$$

(28) $$\bar{v}=-\frac{\partial p}{\partial y}\frac{S}{h/2}=\frac{1}{h/2}\int_{0}^{h/2}v\;dz$$

The combination of the mass equation, momentum equation, and equations for velocity components yields the following equation, which accounts for pressure and fluidity *S*, and is commonly referred to as the pressure drop equation [33,35].

The substituted fluidity *S* is obtained as follows [31,34]:

(29) $$S=\int_{0}^{h/2}\rho \frac{z^{2}}{\eta }\;dz$$

The energy equation is coupled with the pressure drop equation through the temperature-dependent fluid viscosity that needs to be integrated with the fluidity. The energy equation consists of term 1 accounting for the local temporal change of convective heat transfer in the *x* and *y*-directions, term 2 for heat conduction, and term 3 for dissipation and induced shear heating, cf. [36]. In the energy equation, $c_{p}$ represents the specific heat capacity, *T* denotes the temperature, λ is the thermal conductivity coefficient, and $\dot{\gamma }$ represents the shear rate [4,31].

(30) $$\underset{1}{\underbrace{\rho c_{p}\left(\frac{\partial T}{\partial t}+u\frac{\partial T}{\partial x}+v\frac{\partial T}{\partial y}\right)}}=\underset{2}{\underbrace{\lambda \frac{\partial^{2}T}{\partial z^{2}}}}+\underset{3}{\underbrace{\eta {\dot{\gamma }}^{2}}}$$

To provide a concise summary, the Hele–Shaw model equations are formulated based on several simplifications, including the assumption of inelastic flow, neglecting body and inertial forces, disregarding fountain flow phenomena, negligible normal stresses, constant conductivity and heat capacity, and omitting thermal convection in the direction and conduction of the gaps in the flow direction [4]. The presented formulation of the Hele–Shaw model is a widely used modeling approach for flow in injection molding at the midplane. It has become a standard model for research and commercial software applications [2,37,38,39,40]. Some researchers have extended or adapted this approach to simulate various process contents, including fiber orientation [41,42], packing phase [31,32], mold cooling [43], residual stresses [44,45], shrinkage and warpage [46,47], as well as various specialized molding processes. These specialized injection molding processes comprise, e.g., co-injection molding [48], gas-assisted injection molding [49], plastic transfer molding [50], encapsulation of microchips [51], and injection compression molding [40].

### 2.3. 2.5-Dimensional Model

**Figure 4:**
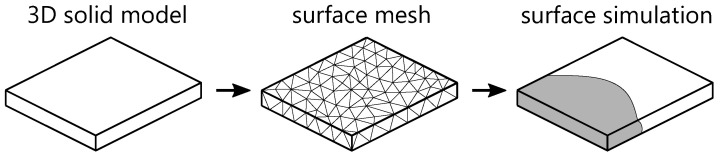
Schematic process of a surface simulation.

The 2.5D model can be described as a surface model for the filling simulation. It represents a 3D part where a skin or topological boundary is generated on the outer surfaces of a solid geometric model. In contrast to midplane models, surface models allow direct analysis of geometric models of thin-walled parts without generating a midplane. Zhou et al. [28,52,53] implemented the first mathematical model. Currently, the surface model is frequently used in commercial injection molding simulation systems [6,54,55,56]. The procedure for applying the surface model is illustrated in Figure 4 [28].

**Figure 5:**
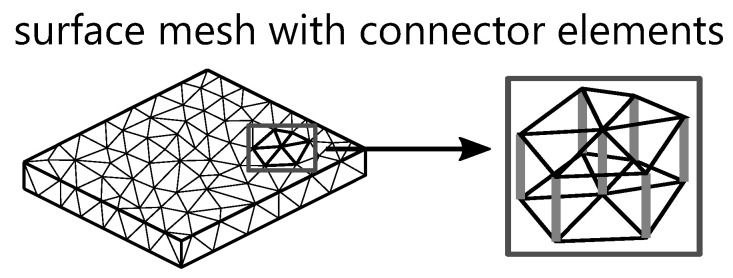
Schematic structure of a surface mesh with connector elements.

In the surface model, the elements on opposite surfaces of a wall are matched and aligned with each other [2,57]. Flow and thermal calculations are then performed on these surfaces [3,55]. To increase the accuracy of this model, connector elements can be included as shown in Figure 5. This is necessary when flow conditions on opposite surfaces do not match. The connector is an additional element that connects the mismatched point through the wall thickness to the other side [2,27,55,57]. This connection between the two surfaces is called dual-domain analysis, where separate analyses are performed for each surface individually.

**Figure 6:**
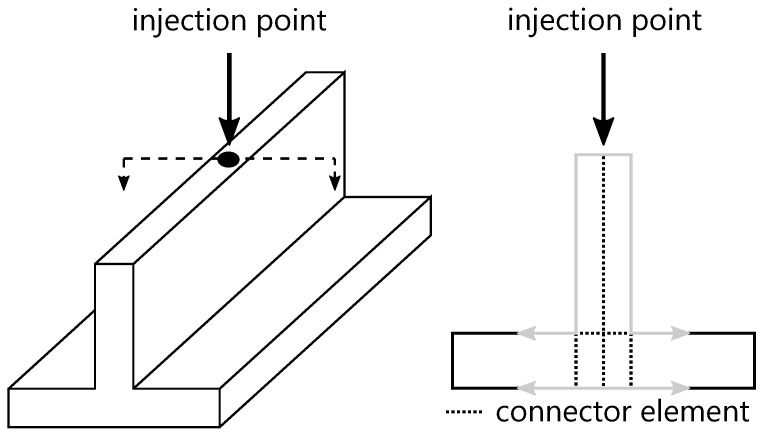
Schematic flow analysis for a part with connector elements.

As real parts are usually more complex than simple plates, Figure 6 shows a schematic component with a rib. In this part the flow in the corner area is transferred to the opposite surface via connector elements [27,58].

It is important to emphasize that the surface model can in principle be converted into a 2D mesh model. This is why the Hele–Shaw approximation is often used for surface models, which is very similar to the midplane model. In combination with the 1D connector elements, the filling simulation can be approximated in three dimensions.

### 2.4. 3-Dimensional Model

The 3D model is based on the full volumetric solid model. The advanced development of computer hardware has made a full 3D simulation of the filling phase in injection molding a reasonable and promising solution to these problems. In comparison to the Hele–Shaw approach, 3D simulation is suitable for complex geometries that do not conform to the criteria of thin-walled designs. This volumetric model is particularly well-suited for thick and massive parts, as well as those with extreme thickness variations [2].

The full volumetric solution of this model is based on the discretization of the Navier–Stokes equations [59]. Assuming that the polymer melt is incompressible during the filling process, the conservation of mass and momentum equations can be derived as follows [60].

(31) $$\frac{\partial }{\partial t}\rho +\nabla \cdot \rho u=0$$

(32) $$\frac{\partial }{\partial t}\left(\rho u\right)+\nabla \cdot \left(\rho \mathrm{uu}-\tau \right)=\rho g$$

These equations contain time *t*, fluid density ρ, velocity field u, gravity *g*, and stress tensor τ. The energy equation (conservation of energy) is defined as follows:

(33) $$\rho c_{p}\left(\frac{\partial T}{\partial t}+u\cdot \nabla T\right)=\nabla \cdot \left(\lambda \nabla T\right)+\eta {\dot{\gamma }}^{2}$$

In this equation, λ is the thermal conductivity, *T* is the temperature, and $\dot{\gamma }$ is the shear rate tensor. The first and second terms represent the rate of change of the energy of fluid particles and the net energy transfer rate by convection, respectively. The first term on the right-hand side of the equation denotes the net heat input to the fluid due to heat conduction, while the second term represents the energy increase due to viscous dissipation [4].

### 2.5. Rheological Model

Polymers exhibit complex rheological properties that significantly influence their flow behavior and processing characteristics. These properties, such as pressure, temperature, and shear rate, can vary considerably. Therefore, selecting an appropriate rheological model is crucial to accurately predict the flow behavior of molten polymers in injection molding simulation. Rheological models are mathematical expressions based on fundamental principles of fluid mechanics that describe the relationship between stress, strain, and rate of deformation of the polymer melt [61]. These models are useful for predicting the filling and packing stages of the injection molding process and the molded part’s final properties. This chapter discusses the most common rheological characterizations of the flow properties of polymer melts. One widely used rheological model in injection molding simulation is the Power-Law model, which assumes a Power-Law relationship between the shear stress and shear rate of the polymer melt. This model is straightforward and easy to implement, making it a popular choice for industrial applications. The second-order viscosity model effectively models critical features of polymer melts such as the convergence of iso-shear rate curves, the convergence of isotherms, and the rate of viscosity decrease with respect to shear rate and temperature [62]. Nonetheless, the model may introduce rheologically implausible behavior, which requires careful consideration during experimental data fitting. The Herschel–Bulkley model is a more complex rheological model that incorporates both the Power-Law and yield stress behavior of polymers. It is commonly used for materials with a substantial yield stress, such as suspensions and gels. The Bird–Carreau model is a non-Newtonian fluid model that characterizes the flow behavior of shear-thinning fluids using a mathematical equation with three adjustable parameters and is particularly useful for materials with high viscosity and shear rates, such as polymer melts and suspensions. The Bingham plastic model is another commonly used model for predicting the flow behavior of viscoelastic materials. This model assumes a linear relationship between the shear stress and shear rate of the polymer melt, with yield stress that must be overcome before the material begins to flow. In addition to these models, other factors, such as temperature shift factors, can be incorporated into the simulation. These factors account for the effect of temperature on the viscosity of the melt. The Carreau and Cross models are frequently used in injection molding simulation to account for the temperature and shear rate dependence of the viscosity of the melt, respectively.

#### 2.5.1. Power-Law Model

The Power-Law model for describing the flow behavior of polymers can be represented by the following mathematical formulations:

(34) $$\eta \left(\dot{\gamma }\right)=\eta_{0}{\dot{\gamma }}^{n-1}$$

(35) $$\eta_{0}=B\;\exp\left(\frac{T_{b}}{T}\right)$$

In this context, $\eta_{0}$ is defined as the Newtonian viscosity or the viscosity at zero shear rate. The constant *B* is referred to as the consistency index, while $T_{b}$ is a constant indicating the temperature sensitivity of the material [63]. *T* denotes the melting temperature, and *n* is the Power-Law index with a value between 0 and 1 for polymer melts. If $\eta_{0}=\mu$ and n=1, the equation for a Newtonian fluid is obtained. The following equation gives the effective shear rate $\dot{\gamma }$ [62,64].

(36) $$\dot{\gamma }=\sqrt{\frac{{\dot{\gamma }}_{ij}^{2}}{2}}$$

The effect of temperature and pressure on viscosity is accounted for by various models, which include the viscosity at zero shear rate [65]. In the context of injection molding, two popular models are commonly employed: the Arrhenius model (also referred to as the five-constant model) and the Williams–Landel–Ferry (WLF) model (also known as the seven-constant model). The Arrhenius model is defined as follows, cf. [66,67,68]:

(37) $$\eta_{0}=B\;\exp\left(\frac{T_{b}}{T}\right)\;\exp\left(\beta p\right)$$

This model is suitable for semi-crystalline materials, where *B*, $T_{b}$, and β are material parameters [69,70]. The WLF viscosity model with the zero-shear rate can provide a more accurate description of the variation of melt viscosity with temperature and pressure [71,72]. This model can be mathematically described as follows:

(38) $$\eta_{0}=D_{1}\;\exp\left(\frac{-A_{1}\left(T-\left(D_{2}+D_{3}p\right)\right)}{A_{2}+T-D_{2}}\right)$$

The constant material parameters $A_{1}$, $A_{2}$, $D_{1}$, $D_{2}$, and $D_{3}$ are included in the equation. By applying the natural logarithm to both sides, Equation (34) can be expressed in the following form:

(39) $$\ln\left(\eta \right)=\left(n-1\right)\;\ln\left(\dot{\gamma }\right)+\;\ln\left(\eta_{0}\right)$$

The three-parameter model presented in this equation accurately determines the behavior of the viscosity function of polymer melts at medium to high shear rates, which is observed to exhibit linearity in a logarithmic representation [62]. Therefore, the Power-Law model can represent the behavior of polymer melts in the high shear rate regime [70]. It is also feasible to fit experimental data with this model and determine the constants $\eta_{0}$ and *n*. The main disadvantage of this model is in the low shear rate range. Despite this disadvantage, the model is often used to model flows in injection molding technology. Particularly in the filling phase, shear rates are often high enough to justify the use of the first-order model [62,73].

#### 2.5.2. Second-Order Model

The second-order model was developed by Moldflow to enhance viscosity modeling in the low shear rate range. This model can be mathematically represented by the following relationship [74].

(40) $$\ln\left(\eta \right)=A+B\;\ln(|\dot{\gamma }\left|\right)+CT+D\;\ln(|\dot{\gamma }{\left|\right)}^{2}+E\;\ln(|\dot{\gamma }\left|\right)T+FT^{2}$$

The presented equation includes material parameters *A*, *B*, *C*, *D*, *E* and *F*, as well as viscosity η, shear rate $|\dot{\gamma }|$ and temperature *T*.

Koszkul and Nabialek [62] clarified in their research the essential contents of the second-order model. While the second-order model can accurately characterize certain behaviors of polymer melts, such as the convergence of iso-shear rate curves with increasing temperature, the convergence of isotherms with increasing shear rate, the decreasing viscosity with increasing shear rate, and the decreasing viscosity with increasing temperature, the authors note that this model is not without its limitations. A major disadvantage is the possibility of introducing behavior that is not rheologically plausible. For example, the derivative of viscosity with respect to shear rate $(\partial \eta /\partial \dot{\gamma })>0$ may suggest that viscosity increases as the shear rate increases, which is unrealistic [75].

#### 2.5.3. Herschel–Bulkley Model

The Herschel–Bulkley model is a commonly used non-Newtonian fluid model that is particularly useful in describing the behavior of fluids with a yield stress, such as the Bingham fluid, but that also exhibit shear thinning behavior. This model can be expressed as the following equation [76,77].

(41) $$\tau =\tau_{0}+m{\dot{\gamma }}^{n}$$

(42) $$\eta =\frac{\tau_{0}}{\dot{\gamma }}+m{\dot{\gamma }}^{n-1}\;\;\mathrm{if}\;\tau >\tau_{0}$$

This model includes yield stress $\tau_{0}$, consistency index *m*, and Power-Law or flow index *n*. As with the Bingham model, the Herschel–Bulkley model requires a certain threshold of stress $\tau_{0}$ to initiate flow. Below this threshold ($\tau \leq \tau_{0})$, the material behaves like a solid, allowing it to sustain stress without flow. Above the threshold, the material behaves like a Power-Law fluid, where n<1 indicates shear thinning, n>1 indicates shear thickening and n=1 reduces the model to the Bingham model and represents Newtonian flow above the critical yield stress [77,78,79,80,81].

#### 2.5.4. Bingham Plastic Model

The Bingham model is a commonly used empirical two-parameter model that is utilized to describe the rheological behavior of materials with yield stresses $\tau_{0}$ that prevent the material from flowing until a certain level of shear stress is reached. Bingham fluids can include materials such as polymer emulsions and slurries. Above the yield stress, a Bingham fluid displays flow characteristics similar to those of a Newtonian liquid and can thus be represented as such.

(43) $$\eta =\infty \;\mathrm{or}\;\dot{\gamma }=0\;\;\mathrm{if}\;\tau \leq \tau_{0}$$

(44) $$\eta =\eta_{0}+\frac{\tau_{0}}{\dot{\gamma }}\;\;\mathrm{if}\;\tau >\tau_{0}$$

In this model, the deviatoric stress tensor is represented by τ, and the dynamic viscosity for zero yield stress is denoted by $\eta_{0}$. The model predicts that a certain threshold of stress must be reached before fluid flow can occur. This critical level of stress is known as yield stress, below which the material remains in a solid-like state [78,79,82,83].

#### 2.5.5. Temperature Shift Factors

The determination of characteristic relaxation times within polymer melts constitutes a fundamental aspect of polymer analysis. However, fluctuations in temperature can induce shifts in time scales. In order to circumvent this issue, alternative models, namely the Arrhenius shift factor and the WLF shift factor, are employed, contingent on the processed material and targeted temperature range. The Arrhenius shift factor model, for instance, serves as a valuable tool in characterizing semi-crystalline polymers, and its mathematical representation is as follows [79]:

(45) $$\alpha_{T}\left(T\right)=\frac{\eta_{0}\left(T\right)}{\eta_{0}\left(T_{s}\right)}=\;\exp\left[\frac{E_{0}}{R_{i}}\left(\frac{1}{T}-\frac{1}{T_{s}}\right)\right]$$

The Arrhenius shift factor involves the zero shear viscosity $\eta_{0}$, activation energy $E_{0}$, reference temperature $T_{s}$, and the universal gas constant $R_{i}$ [84]. This shift allows for the comparison of viscosity curves recorded at different temperatures. By creating a master curve at a specific temperature, viscosity curves measured at various temperatures can be used [79]. For amorphous thermoplastics, the Arrhenius shift factor is valid for temperatures $T>T_{g}+100\;K$ [85,86]. $T_{g}$ refers to the glass transition temperature. Below this temperature, the behavior is dominated by the effects of free volume [87].

Consequently, the temperature dependence of viscosity in amorphous thermoplastics is best described at lower temperatures by the WLF equation [85,88]. This equation describes the viscosity η of the polymer at a specific temperature, *T*, relative to a reference viscosity at a reference temperature, $T_{s}$ [79,84,86].

(46) $$\log\;\alpha_{T}\left(T\right)=\log\;\frac{\eta_{0}\left(T\right)}{\eta_{0}\left(T_{s}\right)}=-\frac{C_{1}\left(T-T_{s}\right)}{C_{2}+T-T_{s}}$$

This equation is applicable to polymers only within the range of zero-shear viscosity. As $T_{g}$ is a commonly used temperature, it is often chosen as the reference temperature $T_{s}$ with $C_{1}=17.44$ and $C_{2}=51.6\;K$. According to [85,89], $T_{g}$ is much lower than typical processing temperatures. Van Krevelen [90] proposes a more appropriate alternative for $T_{s}$, which is $T_{s}=T_{g}+43\;K$, resulting in $C_{1}=8.86$ and $C_{2}=101.6\;K$ [91]. Normally, the viscosity of a polymer is not measured at the reference temperature $T_{s}$, but rather at a temperature within the processing temperature range, denoted as $T^{*}$. This requires a second shift between the measurement or processing temperature $T^{*}$ and the reference temperature $T_{s}$. The following equation represents the relationship between the viscosity at $T^{*}$ and the actual temperature *T* [79,86,92].

(47) $$\log\;\frac{\eta_{0}\left(T\right)}{\eta_{0}\left(T^{*}\right)}=\frac{8.86(T^{*}-T_{s})}{101.6+T^{*}-T_{s}}-\frac{8.86(T-T_{s})}{101.6+T-T_{s}}$$

The equation consists of two terms, where the first term corresponds to the shift between the measurement temperature $T^{*}$ and the reference temperature $T_{s}$. The second term corresponds to the shift between the desired temperature *T* and the reference temperature $T_{s}$.

#### 2.5.6. Carreau Model

The Carreau model is commonly used to describe the flow behavior of polymer flows. It is based on the original research by Bird, Carreau [93,94] as well as Yasuda et al. [95]. From a mathematical point of view, the Carreau model can be described as follows.

(48) $$\eta \left(\dot{\gamma }\right)=\frac{P_{1}}{{\left(1+{\left(\dot{\gamma }P_{2}\right)}^{2}\right)}^{P_{3}}}$$

The three coefficients, $P_{1}$, $P_{2}$, and $P_{3}$, have distinct roles in characterizing the flow behavior. $P_{1}$ can be regarded as zero viscosity, $P_{2}$ represents the transition region between Newtonian and structurally viscous flow behavior, and $P_{3}$ denotes the gradient of the function in the structurally viscous region. All three coefficients are positive, and $P_{3}$ is less than 1 [71,96]. Menges et al. [97] modified this model to incorporate the WLF temperature shift factor $\alpha_{T}$. The equation for the modified Carreau model, also known as the Carreau-WLF model, is described in Equation (49) [98].

(49) $$\eta \left(\dot{\gamma },T\right)=\frac{P_{1}\alpha_{T}}{{\left(1+\alpha_{T}{\left(\dot{\gamma }P_{2}\right)}^{2}\right)}^{P_{3}}}$$

Please note that, for example, in the literature [5,99,100,101], the term ${\left(1+\dot{\gamma }P_{2}\right)}^{P_{3}}$ is often found in the denominator of Equation (48). This is based on the approach that was conceived by Winter [102].

#### 2.5.7. Bird–Carreau Model

The Bird–Carreau model offers a valuable mathematical framework for characterizing the shear rate-dependent behavior of polymer viscosity [68,103]. The model can be represented by the following equation:

(50) $$\frac{\eta -\eta_{\infty }}{\eta_{0}-\eta_{\infty }}={\left[1+{\left(\lambda \dot{\gamma }\right)}^{2}\right]}^{\frac{n-1}{2}}$$

Here, $\eta_{0}$ denotes the viscosity at zero shear rate, λ represents a characteristic time constant, $\dot{\gamma }$ is the shear rate, and *n* represents the Power-Law index. Neglecting the infinite shear rate viscosity $\eta_{\infty }$, the Bird–Carreau model can be simplified to an alternative form [68]:

(51) $$\eta \left(\dot{\gamma },T\right)=\frac{K_{1}\alpha_{T}}{{\left(1+\alpha_{T}{\left(K_{2}\dot{\gamma }\right)}^{2}\right)}^{K_{3}}}$$

In this equation, $K_{1}$, $K_{2}$, and $K_{3}$ denote material-dependent constants, while $\alpha_{T}$ represents a temperature shift factor.

#### 2.5.8. Cross Model

This Cross-model takes into account the effects of shear rate and temperature on viscosity. Similar to the Carreau model, this model describes both Newtonian and shear-thinning behavior. The shear-thinning component is modeled by the general Cross equation [104], which is a popular and an earlier alternative to the Bird–Carreau–Yasuda model [71]. This model is shown in the following equation [79].

(52) $$\frac{\eta_{\dot{\gamma }}-\eta_{\infty }}{\eta_{0}-\eta_{\infty }}=\frac{1}{1+{\left(K\dot{\gamma }\right)}^{1-n}}$$

The parameters used to describe the rheological behavior of the fluid are the zero shear rate viscosity $\eta_{0}$, the infinite shear rate viscosity $\eta_{\infty }$, the time constant *K*, and the Power-Law index *n*, which characterize the fluid’s shear thinning behavior. The cross model can be simplified to the Power-Law model if the fluid has $\eta_{\dot{\gamma }}\ll \eta_{0}$ and $\eta_{\dot{\gamma }}\gg \eta_{\infty }$. If the viscosity is negligible at infinite shear rate, the cross model can be expressed in the following form [101]:

(53) $$\eta_{\dot{\gamma }}=\frac{\eta_{0}}{1+{\left(\frac{\eta_{0}\dot{\gamma }}{\tau^{*}}\right)}^{1-n}}$$

In this equation, $\tau^{*}$ represents the critical shear stress at the transition from the Newtonian plateau. The Power-Law index *n* and the value $K=\frac{\eta_{0}}{\tau^{*}}$ serve as its defining characteristics. The WLF equation can be used to model the zero shear viscosity [105,106,107].

(54) $$\eta_{0}=D_{1}\;\exp\left(\frac{A_{1}\left(T-T_{s}\right)}{A_{2}+T-T_{s}}\right)$$

The viscosity at the reference temperature $T_{s}$ is denoted as $D_{1}$, with $A_{1}$ serving as descriptors of the temperature and $A_{2}$ serving as descriptors of the polymer pressure dependence of the viscosity [108]. This is comparable to the temperature shift factor $\alpha_{T}$ [105,106].

## 3. Overview

### 3.1. Overview of Commercial Software

The development of commercial software for injection molding simulation was strongly dependent on the scientific knowledge and state of the CAD and computer industry at the time. Moldflow was the first company to engage in this type of simulation in 1978 and remains active today. As computers were very expensive in the 1980s, Moldflow’s first products were offered through timesharing services, where users gained access via satellite connections to central computers. The design principles offered by Moldflow were a crucial element of their product, offering users a comprehensive set of guidelines for enhancing the design of polymer parts and gating systems [109]. Early versions of Moldflow software used the “Layflat” approach [110], which reduced the problem of flow in a three-dimensional thin-walled geometry to a two-dimensional plane. Different flow paths were analyzed on the intermediate layer, and the analysis was mainly one-dimensional in terms of pressure drop, although temperature variations were taken into account through the thickness and along the flow path [1,5].

The thermal calculations involved two different methods: one was based on a technique similar to Barrie’s approach [111], which allowed for the determination of the frozen layer’s thickness. The other method employed a finite difference approach, utilizing nodes distributed across both the thickness and the flow path. These approaches assumed a constant mold temperature at the polymer/mold interface. Although the melt temperature at the injection points was assumed to remain constant, the dissipation of viscosity, heat convection caused by the incoming melt, and heat conduction to the mold were taken into account. The viscosity of the melt was modeled using a Power-Law or a second-order model, as used by Williams and Lord [13], considering shear thinning and temperature effects. The pressure drop was determined by employing analytical functions designed for flow calculations in uncomplicated geometries, such as parallel plates or cylindrical pipes. The results of the analysis were presented in tabular form for the flow paths analyzed. The analysis was very fast due to the relatively simple calculation assumptions. By determining pressures and filling times along individual flow paths, the user can increase (or decrease) part thickness to compensate for filling time along individual flow paths and eliminate air inclusion. This type of analysis is undoubtedly beneficial. However, the user had to analyze an abstraction (the Layflat) of the real geometry. It was in 1978 that Giorgio Bertacchi founded the company Plastics & Computer. The distribution of the molding software produced by Plastics & Computer was also facilitated by timesharing systems. These products were focused on all aspects of injection molding. While there were some simulation capabilities, the software also dealt with cost estimation [1,5].

In the 1980s, there was a significant increase in the number of commercially available programs for simulating injection molding. In the early 1980s, Moldflow and Plastics & Computer were the only companies involved in this field. However, by the end of the decade, there were simulation codes available from General Electric (Boston, MA, USA) [112,113,114], Philips/Technical University of Eindhoven (Eindhoven, The Netherlands) [115,116], Graftek Inc. (Austin, TX, USA) [117], Structural Dynamics Research Corporation (SDRC) (Milford, OH, USA) [113], AC-Technology (Fairfax, VA, USA) [118,119], Moldflow Pty. Ltd. (Melbourne, Australia) [110], and Simcon kunststofftechnische Software GmbH (Würselen, Germany). These commercial codes can be categorized into three groups: codes developed by large industrial companies for internal use only, codes developed by large industrial companies for sale on the open market, and codes developed by companies dedicated to the development and sale of simulation codes [1,5,120].

Table 2 provides an overview of currently available commercial software. These differ not only in the numerical models but also in the use of various rheological models. Of particular interest are the commercial software packages that deviate from standard approaches. Cadmould 3D-F uses an advanced 2.5D approach to simulate the filling phase. This differs from the 2.5D approach with surface mesh and connector elements by additionally considering the 2D midplane approach. In this method, the volume is built up with 25 midplanes over the entire part height. These planes are connected by the connector elements. The interpolation between these midplanes allows for a three-dimensional representation of the flow front. The software 3D Timon utilizes a pseudo 3D approach, where the 3D filling process is not solved using the traditional Navier–Stokes equations, but rather the Hele–Shaw approximation method described in Section 2 is extended by a third-dimensional term [121,122]. Compared to a Navier–Stokes solution, this method reduces the number of unknowns at each node from four to two, resulting in significantly lower computational effort [27].

### 3.2. Overview of Research with Commercial Software

The state of the art in an application-oriented context has been influenced by the history of the use of commercial injection molding software. This is based on the research results of the last decades. Today, it is an essential part of the industrial process design of the injection molding process. In addition, these software applications are the source of new research results on the basis of their numerical approaches. Commercial software applications are often used as a substitute model for experiments for the validation of new research approaches. An overview of the current state of research with commercial injection molding software is given in Table 3.

In addition to the dedicated software for the simulation of injection molding processes, research activities are also carried out using commercial CFD software. Non-standard injection molding software includes ANSYS CFX and ANSYS Fluent. These software approaches allow more individualized consideration of boundary conditions and take a fully volumetric (3D) numerical approach. While boundary conditions such as the contact conditions between the melt and the mold wall or surface tensions are fixed in standard injection molding simulations, these can be considered individually in software applications. The implementation of one’s own viscosity models is possible by integrating respective mathematical models [142,143,145,146,147,148,149,150].

### 3.3. Overview of Research Approaches

Injection molding has been a widely utilized process for the production of polymer components for many years prior to the emergence of simulation techniques. Although it was acknowledged that the processing greatly influenced the quality of the end product, the intricate interplay among multiple factors rendered injection molding a craft that relied exclusively on experience to resolve any challenges encountered during the procedure. Rubin [151] provides an overview of this approach, including references to numerous empirical studies that explored the relationship between processing conditions and part quality. [5] Spencer and Gilmore [152,153,154] were pioneers in the study of injection molding processes. Their work, which began in 1950, was focused on the visualization of flow during the mold-filling stage, as well as the modeling of fluid mechanics and the characterization of pressure–temperature variations throughout the molding cycle. Additionally, they investigated the orientation and residual stresses that were present in molded parts. Ballman et al. [155,156,157,158,159] advanced the field by introducing a second generation of studies focused on the non-isothermal modeling of filling rectangular cavities characterized by small thicknesses. Their contributions included the development of the first computer application capable of predictive calculations. Toor et al. [158] proposed a novel approach for determining the mean velocity of a polymer melt during the process of filling a rectangular cavity at a low temperature. They also made an estimation of the maximum flow distance of the polymer. From these calculations, the time required to fill a cavity of a given length could be deduced. The authors integrated convectional heat loss and empirically derived variables for temperature and shear rate impact on viscosity in their computations. Nonetheless, the study did not consider the effects of viscous dissipation, and the pressure equations were derived from force balance. The study’s simulations were computed on an IBM 702 computer, taking approximately 20h/simulation on average [1,5].

The demand for improved quality of molded parts during the 1970s resulted in an increased interest in the mathematical modeling of injection molding processes. Table 4 provides an overview of some published research activities from this decade. Many groundbreaking studies were published during this period. Although in the early seventies, some mathematicians showed interest in Hele–Shaw flow [16], these works were focused on mathematical issues and did not apply to injection molding. In 1971, Barrie [111] analyzed the pressure drop in both the delivery system and a disk cavity. He avoided the need for temperature calculations by assuming a uniform thickness of the frozen layer, which is proportional to the square root of the filling time. Interestingly, Barrie pointed out that the prediction of cavity pressure in the region near the sprue might require tensile viscosity due to the extension rate there. To date, no commercial package includes such terms in the cavity, although pressure losses at sudden contractions, such as gates, are often included [75]. Kamal and Kenig’s [7] work was particularly noteworthy, as they considered the filling, packing, and cooling phases in their analysis. They used finite differences to solve for the pressure and temperature fields. Harry and Parrott [14] and Williams and Lord [13] analyzed the runner system using the finite difference method, which was then extended to analyze the cavity in the filling phase using finite differences [12,27]. Established in 1974, the Cornell Injection Molding Program (CIMP) prompted a shift towards scientific principles in injection molding research [1,5]. Initially, research focused on the filling stage, as demonstrated by Stevenson et al. [160]. The program’s impact on simulation techniques proved influential for both academic and commercial fields [5]. All the previous research focused on rather simple geometries and while it had academic value, it offered limited practical assistance to engineers working with injection molding. However, this research laid the groundwork for the development of commercial simulation tools. In addition to molding simulation research, the 1980s also saw the emergence of computer-aided design (CAD) as a mainstream tool in product design [161]. Early CAD systems were predominantly surface or wire-frame-based, which meant that geometry was represented by surfaces with no thickness displayed [162]. However, the local thickness information has been assigned to the elements during mesh generation [163]. The first technique employed in order to solve the Navier–Stokes equations in these simulations was the finite element method (FEM). This considered a 3D approach to simulate the filling phase. Rajupalem et al. [164,165] used the equal order velocity-pressure formulation to describe the mold filling and packing phases, which was also used in other similar research studies [166,167]. Haagh et al. [60] implemented a pseudo-concentration method in their finite element program for injection molding filling. In addition, Haagh et al. [168], Zhou et al. [169], and Ilinca et al. [100] used the finite element method to solve the Navier–Stokes equations in their 3D numerical model for gas-assisted injection molding. Classical Galerkin formulations of viscous incompressible Navier–Stokes equations can result in spurious numerical oscillations, leading to failure in finite element methods [170]. To overcome this issue, the Petrov-Galerkin method has been widely utilized in the solution of coupled equations of flow and heat transfer [171,172,173,174,175]. This method has also been employed to prevent potential numerical instabilities [176,177]. Chang and Yang [178] developed a numerical simulation program for mold filling using an implicit finite volume approach (FVM). This was later extended to cover various stages of injection molding and other molding processes [179,180]. Similar approaches have been employed by Zhou and Turng [181], Chau and Lin [182], and Seo et al. [183,184]. The control-volume-based finite-element-method (CVFEM) combines the advantageous properties of both finite element methods and finite volume methods. This method utilizes the flexibility of the FEM to discretize complex geometries with the conservative formulation of the FVM, which allows for easy physical interpretation of variables in terms of flux, forces, and sources [185,186,187]. Therefore, the CVFEM is a newly employed technique for solving flow and heat transfer in injection molding [188,189]. The boundary element method (BEM) has been extensively used for cooling simulation in injection molding, but its application for solving the 3D flow problem of injection molding is limited [190,191,192]. One way to address the 3D filling simulation is through accurate tracking of melt fronts (polymer/air interfaces) and the evolution of their complex topology. The choice of the reference frame determines whether Lagrangian or Eulerian algorithms are used to tackle moving boundary problems. Lagrangian algorithms move with the fluid, resulting in accurate free-surface representation and easy inclusion of surface tension, but require frequent domain re-meshing and are computationally intensive in 3D [192,193,194]. Eulerian algorithms use a fixed reference frame with a single mesh, allowing interpolation to treat large interface distortions, but are limited by the accuracy of the interface position due to grid size and cannot easily include surface tension [2]. There are several algorithms used in Eulerian analysis, including surface tracking [195], level set [196,197,198], and volume tracking [2]. Among these, volume tracking is the most widely used in injection molding simulation. This method includes popular techniques such as marker and cell (MAC) [199], flow analysis network (FAN) [23], and volume of fluid (VOF) [197,200,201,202,203,204]. In [3], Cardozo presents the underlying principles and methods of free surface tracking in injection molding simulation.

## 4. Conclusions

Numerical approaches offer a wide range of solutions for the simulation of injection molding processes. It is important to understand the advantages and disadvantages of the different approaches and to weigh them up for each individual problem in order to select the right modeling method. For customized solutions, 2D, 2.5D, and 3D models are particularly promising. While 1D models are good for simple geometries, 2D and 2.5D models offer a higher degree of accuracy. It should be noted, however, that they are based on a simplification in the sense of the Hele–Shaw approach and are most suitable for thin-walled geometries. On the other hand, 3D models are very accurate but require more computing time because of the volumetric calculations. Research results in the literature, e.g., [74,147,148,215], show that these models can be sufficiently accurate for the filling phase. A summary of the main differences in the mathematical models is given in Table 5 [220].

It should be noted that not all viscosity models cover all relevant variables when selecting a viscosity model. These influencing variables can be divided into three categories: shear rate, temperature, and pressure. An overview of the variables associated with each viscosity model is given in the Table 6, but it should be noted that the inclusion of additional variables will increase the complexity of the calculation. Viscosity models have undergone numerous modifications over time, as described in the literature, e.g., [108]. Polymer flow behavior is very similar to unmodified viscosity models, so no further distinction is made between these different approaches.

Several approaches have been investigated over the decades with respect to spatial approximation. The most widely used viscosity model in both research and commercial applications is the Cross model with appropriate modifications. However, due to increased computing power, there has been a recent focus on 3D models. In the commercial sector, 2D and 2.5D models are still widely used. The reason for this is that they are faster to calculate. Both the Hele–Shaw model and full volumetric solutions are now offered by some suppliers. This can be attributed to the neglect of flow in the melt front (fountain flow), the viscous convection (drag force), the heat conduction at the lateral wall faces, and the representation of gap solutions at the flow branch and at the wall thicknesses. This is where 3D models offer a clear advantage, as they can resolve the entire filling phase much more accurately without simplifying. This is particularly useful for thicker or more complex parts, where simplifying can produce inaccurate results.

Despite extensive development efforts over a long period of time, numerical approximation of injection molding filling behavior continues to improve. Advancing the modeling of influencing factors within the contact region between the mold and the polymer during the filling process is a notable endeavor. The complex task of faithfully representing the filling dynamics that occur in this region is being addressed through experimental validation of software applications. The accuracy of these models stands to improve by giving greater consideration to boundary influences, exemplified by factors such as mold surface roughness as well as surface tension of the polymer. Another important facet requires adopting a more holistic paradigm when modeling filling simulations. An example of this is the general neglect of inertia and gravitational effects, primarily due to their relatively small influence in comparison to viscous forces, and the complexity of accommodating them within a framework. However, in contexts characterized by intricate, thick-walled components or scenarios involving high velocity flows within planar structures such as ribbed plates, constricting gates, junctions, or abrupt thickness changes, their importance could be magnified and potentially control the course of the filling pattern. Another significant research gap is the treatment of non-standard injection molding software. Often these software systems treat individual regions as uniform “outlets”, without distinguishing between the polymer medium and the surrounding air. This makes analyzing pressure dynamics during filling particularly difficult. To overcome this challenge, an important avenue for progress is the development and subsequent validation of methodologies that accurately distinguish the relevant media components. Finding solutions to these research gaps will contribute significantly to improving the understanding and accuracy of simulating injection molding processes.

## Figures and Tables

**Table 1:** Governing relations for 1D volume flows of different geometry types.

	Center Gated Disc	Tube	Strip
Volume flow $\dot{V}$	$\dot{V}=2\pi R\int_{-h/2}^{h/2}\dot{\gamma }y\;dy$	$\dot{V}=\pi \int_{0}^{R}\dot{\gamma }r^{2}\;dr$	$\dot{V}=b\int_{-h/2}^{h/2}\dot{\gamma }y\;dy$
Shear rate $\dot{\gamma }$	$\dot{\gamma }=\frac{\Lambda y}{\eta }$	$\dot{\gamma }=\frac{\Lambda r}{2\eta }$	$\dot{\gamma }=\frac{\Lambda y}{\eta }$
Pressure gradient Λ	$\Lambda =\frac{\dot{V}}{2\pi RS}$	$\Lambda =\frac{2\dot{V}}{\pi S}$	$\Lambda =\frac{\dot{V}}{bS}$
Fluidity *S*	$S=\int_{-h/2}^{h/2}\frac{y^{2}}{\eta }\;dy$	$S=\int_{0}^{R}\frac{r^{3}}{\eta }\;dr$	$S=\int_{-h/2}^{h/2}\frac{y^{2}}{\eta }\;dy$

**Table 2:** Overview of commercial software applications.

Supplier	Software	Type of Model Approach	Rheological Model	References
Autodesk, Inc. (San Fransico, CA, USA)	Moldflow	2.5D, 3D	Cross-WLF, second-order	[101]
CoreTech System Co., Ltd. (Zhubei City, Taiwan)	Moldex3D	2D, 3D	Power-Law, Cross-WLF, Carreau, Herschel–Bulkley	[5,101,123,124]
C-Solution, Inc. (Boulder, CO, USA)	Simuflow	2D	Carreau	[5,125]
Hexagon AB (Stockholm, Sweden)	VISI-Flow	2.5D (only surface)	Cross-WLF	[5,126]
SIGMA Engineering GmbH (Aachen, Germany)	Sigmasoft	3D	Cross-WLF, Herschel–Bulkley	[5,101,127]
Simcon kunststofftechnische Software GmbH (Würselen, Germany)	Cadmould 3D-F/3D-V	advanced 2.5D, 3D	Carreau-WLF, Cross-WLF	[5,101]
Toray Engineering Co., Ltd. (Tokyo, Japan)	3D TIMON	pseudo 3D	Cross-WLF	[1,5,27,128]
Tanslavor S.A. (Biot, France)	Rem3D	3D	Cross-WLF, Cross-Arrhenius	[1,5,27,129]

**Table 3:** Overview of the current research using commercial software.

Year	Author	Commercial Software	Rheological Model	References
2008	Zhou et al.	Moldflow	Cross-WLF	[130]
2019	Yu et al.	Moldflow	Cross-WLF	[131]
2022	Lin et al.	Moldflow	Cross-WLF	[132]
2023	Yu et al.	Moldflow	Cross-WLF	[133]
2019	Tran and Gehde	Moldex3D	Cross-WLF	[134]
2019	Islam et al.	Moldex3D	Cross-WLF	[135]
2022	Tsai et al.	Moldex3D	Cross-WLF	[136]
2011	Mannella et al.	VISI-Flow, Moldflow	Cross-WLF	[126]
2011	Mulser et al.	Sigmasoft	Cross-WLF	[137]
2013	Ariff and Khang	Cadmould 3D-F	Carreau-WLF	[138]
2017	Ou et al.	Cadmould 3D-F	Carreau-WLF	[139]
2017	Othman et al.	Cadmould 3D-F	N/A	[140]
2020	Sahli et al.	Cadmould 3D-F	Carreau-WLF	[141]
2009	Shin et al.	3D Timon	Cross-WLF	[128]
2005	Silva et al.	Rem3D	Cross-WLF	[129]
2014	Fang et al.	ANSYS CFX	Power-Law-Arrhenius	[142]
2016	Zhuang et al.	ANSYS CFX, Moldflow	Cross-WLF	[143]
2016	Mukras and Al-Mufadi	ANSYS CFX	Newtonian	[144]
2021	Anders et al.	ANSYS CFX, Cadmould 3D-F	Carreau-WLF	[145]
2021	Baum and Anders	ANSYS CFX, Cadmould 3D-F	Carreau-WLF	[146]
2022	Baum et al.	ANSYS CFX	Carreau, Carreau-WLF	[147]
2016	Rusdi et al.	ANSYS Fluent	Cross-Arrhenius	[148]
2022	Zaki et al.	ANSYS Fluent	Cross	[149]
2023	Abdullah et al.	ANSYS Fluent	Cross-Arrhenius	[150]

**Table 4:** Overview of the research solution.

Year	Author	Model Approach	Rheological Model	References
1971	Barie	1D	Power-Law	[111]
1972	Kamal and Kenig	1D	Power-Law	[7,8]
1973	Berger and Gogos	1D	Power-Law	[205]
1973	Broyer et al.	1D	Newtonian	[21]
1974	Wu et al.	1D	Power-Law	[9]
1975	Lord and Williams	1D	second-order	[12]
1975	Williams and Lord	1D	second-order	[13]
1977	Nunn and Fenner	1D	Power-Law	[10]
1978	Stevenson	1D	Power-Law	[11]
1979	Stevenson and Chuck	1D	Power-Law	[206]
1974	Tadmor et al.	2D	Power-Law	[23]
1978	Hieber and Shen	2D	Power-Law	[24]
1980	Hieber and Shen	2D	Power-Law	[25]
1986	Wang et al.	2D	Cross-Arrhenius	[37]
1991	Chiang et al.	2D	Cross-WLF	[31,32]
1993	Chiang et al.	2D	Cross-WLF	[46]
1994	Chen and Liu	2D	Cross-Arrhenius, Cross-WLF	[67]
1995	Chung and Kwon	2D	Cross	[41]
1996	Chung and Kwon	2D	Cross	[207]
1997	Han and Im	2D	Cross-WLF	[208]
1999	Holm and Langtangen	2D	Power-Law	[196]
2007	Estacio and Mangiavacchi	2D	Cross-Arrhenius	[188]
2008	Wang et al.	2D	Cross-WLF	[209]
2017	Xu and Yu	2D, 3D	Newtonian, Cross	[210]
1995	Kwon and Ahn	2.5D	Cross	[211]
1997	Rajupalem et al.	2.5D	N/A	[164]
1998	Talwar et al.	2.5D	N/A	[165]
2001	Zhou and Li	2.5D	Cross-Arrhenius	[52]
2001	Zhou et al.	2.5D	Cross-Arrhenius	[28]
2001	Chang and Yang	2.5D, 3D	Newtonian	[178]
2002	Zhou and Li	2.5D	Cross-Arrhenius	[53]
1998	Hétu et al.	3D	Newtonian, Bird–Carreau, Bird–Carreau–Arrhenius	[176]
1998	Pichelin and Coupez	3D	Bird–Carreau	[212]
1999	Pichelin and Coupez	3D	Bird–Carreau	[213]
1999	Zheng et al.	3D	Cross	[214]
2000	Ilinca and Hétu	3D	Cross-WLF	[215]
2001	Haag et al.	3D	Cross-WLF	[168]
2001	Khayat et al.	3D	Newtonian	[192]
2002	Hwang and Kwon	3D	Cross	[216]
2003	Ilinca and Hétu	3D	Carreau-WLF	[100]
2004	Yang et al.	3D	Cross-Arrhenius	[179]
2005	Cao et al.	3D	Cross-WLF	[217]
2005	Zhou et al.	3D	Cross-Arrhenius	[167]
2006	Kim and Turng	3D	Power-Law, Cross-WLF	[166]
2006	Zhou and Turng	3D	Power-Law	[181]
2012	Wang et al.	3D	Cross-Arrhenius, second-order	[74]
2017	Liang et al.	3D	Cross-WLF	[218]
2017	He et al.	3D	Power-Law	[219]

**Table 5:** Overview of the difference of the mathematical models for the filling phase.

	1D Model	2D Model	2.5D Model	3D Model
Assumptions for a thin-walled laminar flow (Hele–Shaw)	X	*√*	*√*	X
Assumption of incompressibility	*√*	*√*	*√*	*√*
Heat transfer in flow direction	X	*√*	*√*	*√*
Constant physical variables	*√*	*√*	*√*	*√*
Planar flow front	X	*√*	*√*	X
Mesh Size	Coarse	Medium	Fine	Ultrafine
Complexity of numerical model	Very simple	Simple	Complex	Very complex
Solution time	Very short	Short	Moderate	Long

**Table 6:** Overview of viscosity models and their influencing variables.

Viscosity Model	Shear Rate	Temperature	Pressure
Power-Law	*√*	X	X
Second-order	*√*	*√*	X
Herschel–Bulkley	*√*	X	X
Bingham	*√*	X	X
Carreau	*√*	X	X
Bird–Carreau	*√*	X	X
Cross	*√*	X	X
Power-Law-Arrhenius	*√*	*√*	*√*
Carreau-WLF	*√*	*√*	*√*
Bird–Carreau–Arrhenius	*√*	*√*	X
Cross-WLF	*√*	*√*	*√*
Cross-Arrhenius	*√*	*√*	*√*

## Data Availability

Not applicable.
